# Changing dynamic phosphorus forms from field to stream during surface runoff events

**DOI:** 10.1002/jeq2.70096

**Published:** 2025-12-17

**Authors:** Rebecca M. Kreiling, Tanja N. Williamson, Faith A. Fitzpatrick, Kenna J. Gierke, James D. Blount, Patrik M. Perner, Isaac J. Mevis, Heidi M. Broerman, Katherine R. Merriman, Matthew J. Komiskey

**Affiliations:** ^1^ U.S. Geological Survey, Upper Midwest Environmental Sciences Center La Crosse Wisconsin USA; ^2^ U.S. Geological Survey, OH‐KY‐IN Water Science Center Louisville Kentucky USA; ^3^ U.S. Geological Survey, Upper Midwest Water Science Center Madison Wisconsin USA; ^4^ U.S. Geological Survey, Upper Midwest Water Science Center St. Paul Minnesota USA; ^5^ U.S. Geological Survey, Upper Midwest Water Science Center Rhinelander Wisconsin USA; ^6^ U.S. Geological Survey, Upper Midwest Water Science Center Green Bay Wisconsin USA; ^7^ U.S. Geological Survey, New York Water Science Center Troy New York USA

## Abstract

The risk of water quality impairment from agricultural runoff depends on nutrient source, transport, and bioavailability. Phosphorus (P) spirals between dissolved and particulate forms as it is transported with suspended sediment (SS) from agricultural fields, through the stream network, to receiving water bodies. This dynamic sorption‐desorption influences bioavailability. We quantified P form and abundance in samples collected during surface‐runoff events from a farm field in the East River Basin, Wisconsin and compared them to those in stream water collected from the East River. We sampled five events between late March 2022 and June 2023. During most events, P in surface runoff was mainly in dissolved form, with particulate P sorbed to fine clay, the most abundant particle fraction transported from the field, whereas P in stream water was mainly in particulate form and sorbed to silt, even though fine clay was the most abundant particle fraction in the stream during events. Overall capacity for P sorption to SS in the stream varied among events. Total P and SS concentrations were lower during summer baseflow conditions and smaller surface runoff events; however, what SS was present was more P enriched. This shift in P form from field to stream indicates a potential for sorbing dissolved P to SS during transport through the stream network, which changes the bioavailability of P exported downstream with less bioavailable P as dissolved P binds to SS.

## Core Ideas

- During runoff events, most P leaving field was dissolved, whereas at least half of P in stream was particulate.
- Downstream bioavailability of P differed as a function of dissolved‐particulate P exchange.
- SS was more P‐enriched during summer baseflow conditions and during smaller surface runoff events.
- Surface runoff events highlight short‐term effects of on‐field management actions on P runoff from the field.

## Plain Language Summary

Agriculture unintentionally causes phosphorus, a key plant nutrient, to be lost to rivers. Higher concentrations of phosphorus can lead to harmful algal blooms in rivers and lakes. Phosphorus is less likely to be taken up by algae if it is attached to suspended sediment in the river. In this study, we collected water that was running off a farm field during storm events, and at the same time, we collected water in the river downstream of the farm field. Our objective was to quantify the amount of phosphorus that was in the dissolved form or attached to suspended sediment in samples collected at both sites. We found that during most events, water leaving the field contained phosphorus that was mainly in the dissolved form, whereas at the river site, phosphorus was mainly attached to fine suspended sediment particles. This means that the phosphorus in the water at the river site is less likely to be taken up by algae, potentially leading to a lower likelihood of an algal bloom occurring.

**Abbreviations**
DRP: dissolved reactive phosphorus
EPC_0_
: equilibrium phosphorus concentration
HAB: harmful algal bloom
PP: particulate phosphorus
SS: suspended sediment
TP: total phosphorus
USGS: United States Geological Survey

## 1 INTRODUCTION

Excess nitrogen (N) and phosphorus (P) inputs from agricultural land use have caused eutrophication of freshwater and coastal areas, leading to harmful algal blooms (HABs) and hypoxia (Rabalais & Turner, 2019; V. H. Smith et al., 2006; Wurtsbaugh et al., 2019). Blooms occur in all five Laurentian Great Lakes (Bosse et al., 2024), with greater prevalence in shallow, eutrophic bays that receive agricultural runoff (Sayers et al., 2016). Management of nutrient pollution in agricultural areas has focused on on‐field conservation practices designed to limit soil erosion and nutrient loss during runoff events (Macrae et al., 2021; Moriasi et al., 2020) when most sediment and nutrient loading occurs (Dolph et al., 2019; Lloyd et al., 2016; Ockenden et al., 2016). Yet, even in fields with multiple conservation practices, soil and nutrients are still lost (Moriasi et al., 2020; Sharpley et al., 2009).

Phosphorus is typically the growth‐limiting nutrient in freshwater ecosystems, and efforts to reduce P runoff are included in management actions (Sharpley et al., 2009). Total P (TP) in freshwater consists of dissolved inorganic (reactive) P (DRP), dissolved organic P, and particulate P (PP) and colloidal P, which are bound to suspended sediment (SS). Conservation practices that target soil erosion and improve soil health are also intended to decrease PP loss (USDA‐NRCS, 2020). However, some on‐field conservation practices, such as conservation tillage, can inadvertently increase loss of DRP due to soil P stratification and accumulation, increased leaching through preferential flow pathways, and P leaching from decaying plants (Duncan et al., 2019; Jarvie et al., 2017; Macrae et al., 2021). In some western Lake Erie tributaries, concentrations of DRP are increasing as TP concentrations are decreasing or remaining unchanged (Rowland et al., 2021).

Understanding and managing cropland soil‐P helps minimize P loss (K. W. King et al., 2018); however, it is also important to know how P is transported from the field, including the form (dissolved or particulate) and particle sizes of SS transporting sorbed PP. The P form leaving the field can impact downstream aquatic ecosystems because not all P is bioavailable. Dissolved P is almost entirely bioavailable (Ellison & Brett, 2006), whereas PP bioavailability in Great Lakes agricultural tributaries ranges from 25% (Baker et al., 2014) to 89% (Lin et al., 2018). PP bioavailability is dependent on the physical and chemical properties of the accompanying SS. Loosely sorbed PP, labile organic PP, and P bound to metal hydroxides present on SS, such as redox‐sensitive iron and manganese, are all biologically labile (James & Larson, 2008). Chemical processes such as de/sorption and redox affect bioavailability of these labile P forms (Withers & Jarvie, 2008). SS particle size also can affect bioavailability. Fine‐grained particles (<63 µm) have a greater specific surface area to adsorb P and are readily transported during runoff events (Poirier et al., 2012; Sharpley et al., 2013).

After P leaves the field, it actively cycles between dissolved and particulate forms as it moves through the stream network, changing the ratio of DRP to TP (DRP:TP) and thus the pool of bioavailable P (W. M. King et al., 2022; Withers & Jarvie, 2008). PP can be deposited and stored in streambed sediment (Williamson et al., 2024). Dissolved P can be assimilated through biotic uptake (Larson et al., 2019) or de/sorbed from SS (W. M. King et al., 2022; Williamson et al., 2023) and streambed sediment (Kreiling et al., 2023). Sorption of P to SS does not change TP concentration but does reduce bioavailable P (W. M. King et al., 2022), potentially slowing downstream transport of bioavailable P. The magnitude of P de/sorption to SS is determined by concentrations of SS, DRP, and PP, which combine to govern the dynamic P equilibrium between SS and stream water (Froehlich, 1988). In Great Lakes tributaries, PP enrichment in SS commonly exceeds that in streambank, streambed, and upland sources of sediment (Blount et al., 2022; Fitzpatrick et al. 2019; Williamson et al., 2024).

Our objective of this study was to compare the interaction of P with SS in field surface runoff relative to the stream. We characterized P forms and abundance in surface runoff sampled at a farm field and stream water collected at the upper East River United States Geological Survey (USGS) streamgage (Site ID 04085108; Figure S1). Included in our PP analysis at each location was assessment of P abundance among dissolved and particulate fractions. We hypothesized (i) that most PP at both locations would be in the fine clay fraction (<1 µm) of SS and (ii) that DRP:TP would differ between the field and East River, with a higher proportion of DRP at the field due to conservation practices that were implemented to reduce soil erosion.

## 2 SITE DESCRIPTION

We sampled at the upper East River USGS streamgage, which drains 116 km^2^ of the 376 km^2^ East River Basin. The East River drains into the Fox River, at Green Bay, Wisconsin. The Fox River contributes approximately 22% of the annual TP load to Lake Michigan (Robertson et al., 2019) and is listed as a priority watershed for nutrient reduction by the US Environmental Protection Agency (Merriman et al., 2019). The predominant land use in the Fox River Basin is agriculture (Figure S1; Dewitz & U.S. Geological Survey, 2024), including row crops and pasture. Permitted confined animal feeding operations (CAFOs), where animals have minimal grazing outside, are scattered throughout the basin (Kreiling et al., 2018). Liquid manure from farms is used as fertilizer for agricultural fields (Kreiling et al., 2018; Merriman et al., 2019). The upper East River subbasin has seven dairy cow CAFOs (https://dnr.wisconsin.gov/topic/CAFO/StatsMap.html). Conservation practices have been implemented throughout the subbasin to reduce nutrient and sediment runoff from the fields (Kreiling et al., 2018; Merriman et al., 2019). From 2012 to 2021, mean annual yields of SS, TP, and DRP from the upper East River were 485 kg·ha^−1^·year^−1^, 1.66 kg P·ha^−1^·year^−1^, and 0.77 kg P·ha^−1^·year^−1^, respectively (Table S1; U.S. Geological Survey, 2024); this indicates a DRP:TP of 0.47, or approximately half, over the period of record at the stream site.

**Core Ideas**

- During runoff events, most P leaving field was dissolved, whereas at least half of P in stream was particulate.
- Downstream bioavailability of P differed as a function of dissolved‐particulate P exchange.
- SS was more P‐enriched during summer baseflow conditions and during smaller surface runoff events.
- Surface runoff events highlight short‐term effects of on‐field management actions on P runoff from the field.

We also sampled a 2.87‐ha farm field (USGS station 441520088045001; Figure S1) ∼31 km upstream of the streamgage that does not discharge directly into the stream network. Implementation of on‐field conservation practices began extensively in November 2016 (Hanrahan et al., in press). This field has 8 m of relief (Webber & Williamson, 2021) and was planted in a corn rotation through 2020, then wheat (2021), corn silage (2022), and alfalfa (2023). The field is fertilized with a combination of manure and chemical fertilizers, has subsurface (tile) drainage, and is occasionally tilled in the spring or fall using chisel and disk implements (Fermanich et al., 2023; Komiskey et al., 2021). The last tillage of the field before the study commenced occurred in May 2020 (Gierke et al., 2025). Beginning in 2016, the field was planted with cover crops during the non‐growing season (Hanrahan et al., in press). During winter 2022/2023, the cover crop did not grow well, with soils bare through early spring 2023.

Soils at the field are the Waymor silt loam series with mean organic matter content of 4.0% and mean soil test‐P (Bray‐P) and TP concentrations of 80 and 904 mg P·kg^−1^, respectively, in the top 5 cm of soil (Meyers et al., 2021). Soils freeze during the winter, but surface runoff and tile discharge still occur during frost conditions. Mean annual surface runoff yields of SS, TP, and DRP measured at the field from 2014 to 2021 were 2393 kg·ha^−1^·year^−1^, 6.92 kg P·ha^−1^·year^−1^, and 2.09 kg P·ha^−1^·year^−1^, respectively (Table S1; U.S. Geological Survey, 2024). Mean annual SS and P yields from the monitored tile from 2014 to 2021 were 56.8 kg·ha^−1^·year^−1^ for SS, 0.39 kg P·ha^−1^·year^−1^ for TP, and 0.26 kg P·ha^−1^·year^−1^ for DRP. Surface runoff is 11%–33% of total runoff from field, but it is 88%–98% of SS annual load and 81%–99% of TP load among years.

**FIGURE 1:**
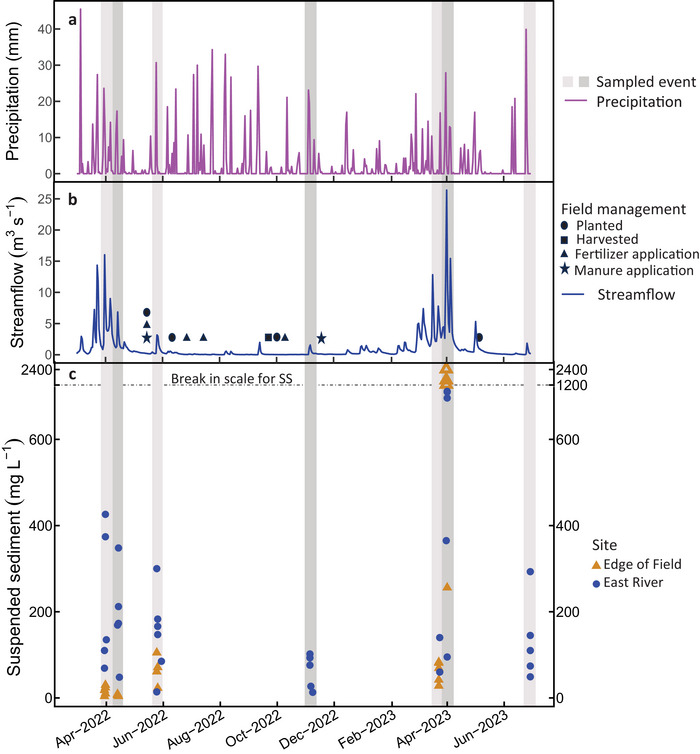
Precipitation for the study period measured at the Green Bay Austin Straubel International Airport weather station (a). Streamflow measured at the East River site and field management actions at the farm field site (b). Concentrations of suspended sediment (SS) in water samples collected during surface runoff events (c). Note the three open triangles depict SS samples at the field that had concentrations above the *y*‐axis break. Each individual event is highlighted by a separate shaded gray line.

Total precipitation measured at the Green Bay airport during the 16‐month study period was 1226 mm (MRRC, 2024; Figure 1a). Snowfall total during November 2021–March 2022 was 1057 mm (244‐mm snow‐water equivalent), whereas during November 2022–March 2023, it was 1872 mm (305‐mm snow‐water equivalent). Mean annual precipitation in the study area is 741 mm from 135 years of data (Midwest Regional Climate Center [MRCC], 2024). Streamflow was low for most of the study, with elevated streamflow only observed during March and April of both years (Figure 1b). Mean frost depth during winter of 2021/2022 was 711 mm, with maximum depth of 1041 mm measured on March 17, 2022. Mean frost depth during winter of 2022/2023 was 89 mm, with maximum depth of 356 measured on February 2, 2023 (National Weather Service [NWS], 2025).

## 3 METHODS

### 3.1 Collection of samples

Streamflow and water quality sampling began in the East River in December 2011 and at the field in March 2014. Continuous, 15‐min interval, streamflow measurements were recorded at the gage during baseflow and manually adjusted to 1‐min intervals during events. Surface runoff at the field was monitored using an H‐flume to direct and quantify runoff (Stuntebeck et al., 2008). Automated refrigerated water samplers collected event water at the gage and surface runoff from the field. Although tiles contribute to total loads leaving the field, tiles were only monitored from March 2014 to September 2021 and were not included in this study.

Sample collection for this study occurred from March 2022 to June 2023. Four streamflow events were sampled in 2022, with field surface runoff only generated in the first three events, and three streamflow events were sampled in 2023, with field surface runoff only generated in the first two events (Table S1; Figure 1). Samples were not collected for particle size analysis during all events but were still collected to measure DRP concentrations.

A total of five discrete samples were collected across the hydrograph at both field and stream for each event, with samples collected during the rising limb, peak, and recession of the hydrograph. Duplicate samples were collected at each time point. One sample from each time point was analyzed for SS, TP, and DRP concentrations. The East River SS, TP, and DRP loads were computed using these discrete sample results and the East River streamflow record using the Graphical Constituent Loading Analysis System (GCLAS; Koltun et al., 2006). To estimate yearly loads, daily loads calculated with the GCLAS method were summed across the annual period of record. At the field, flow‐weighted mean concentrations were computed for each surface runoff event and multiplied by surface runoff volume to calculate loads. Event yield was then calculated by dividing the load by the field drainage area. Total PP concentration was normalized for SS (P_ss_ = [TP − DRP]/SS; Williamson et al., 2023). We also collected two baseflow samples at the stream site in August 2022 to assess if the same sediment dynamics were occurring during baseflow as observed during runoff events.

### 3.2 Measurement of P in particle‐size fractions

The replicate for each sample was analyzed within 48 h to measure DRP and PP concentrations in different particle‐size fractions of the SS in stream water and field surface runoff. Samples were split into various particle‐size fractions using centrifugation based on methods outlined in Jackson (2005) and then analyzed for P (American Public Health Association [APHA] et al., 2017). Subsamples were first collected for TP and total sediment mass. The sand (>63 µm) fraction was separated by settling, and then samples were collected to determine sediment mass and PP amount in the remaining fine‐grained (<63 µm) fraction. The silt and coarse clay (1–63 µm) fraction was separated via centrifugation, and samples were collected to determine sediment mass and PP amount in the remaining fine clay (<1 µm) fraction. We collected a DRP sample from the fine‐clay fraction filtrate using a 0.45‐µm filter (American Public Health Association [APHA] et al., 2017). After the 2022 sampling season, centrifugation was refined to include both the coarse clay (1–2 µm) and fine clay (<1 µm) fractions. Thus, in 2022, we determined P in whole water, sand (>63 µm), silt and coarse clay (1–63 µm), fine clay (<1 µm), and dissolved fractions; in 2023, we determined P in whole water, sand (>63 µm), silt (2–63 µm), coarse clay (1–2 µm), fine clay (<1 µm), and dissolved fractions.

### 3.3 Measurements of SS P dynamics

Flow‐integrated SS samples were collected from April 18 to November 14, 2022, at the stream site using two methods. Passive, in‐situ PVC‐sampling tubes (Phillips et al., 2000) were deployed 0.2 m above the streambed (approximately 0.6 times mean water depth) near the thalweg, to collect a weekly representative SS sample. SS samples were aggregated as monthly to bimonthly samples depending on SS volumes. Samples were centrifuged immediately after retrieval using a streamside centrifuge to dewater the sediment. The second collection method involved using the sampler head from an automated ISCO pump. The ISCO intake tube was located approximately 3 m off the left bank about 0.10 m above the streambed and was triggered to create a flow‐based sample that represented both baseflow and stormflow periods. All sampled water was pumped into a 55‐gallon bucket that was in an insulated, aluminum shed along the stream, which kept the water at ambient temperature. The water in the bucket was centrifuged weekly. Weekly samples were composited based on time frames of the passive sample deployment, so the two different methods could maintain the same deployment range for each composited sample.

The equilibrium P concentration (EPC_0_), which is an index of the potential for sediment to de/sorb P, was determined following methods reported in Kreiling et al. (2023) for at least one of the two SS samples collected each month based on the amount of sediment that was collected. The EPC_0_ is the DRP concentration in stream water at net zero sorption of P to the sediment. For each SS sample, six 50‐mL centrifuge tubes, each containing 1.5‐g wet sediment, were spiked with six different concentrations of P (2.0, 1.0, 0.5, 0.1, 0.05, and 0 mg P·L^−1^). Samples were shaken in the dark for 24 h at room temperature and then centrifuged. The supernatant was filtered and analyzed for DRP. The EPC_0_ was determined by fitting amount of P sorbed (mg P·kg dry per sediment^−1^) against final DRP concentration (mg P·L^−1^) using the Langmuir adsorption isotherm and then calculating the x‐intercept, which denotes the EPC_0_ (Simpson et al., 2021). All measured stream‐water DRP concentrations collected during the study period, including samples collected for routine USGS monitoring (U.S. Geological Survey, 2024), were compared to estimated EPC_0_ concentrations to assess if SS was adsorbing or desorbing P. Additionally, to assess the P amount in the SS that has the potential to be released during transport, MgCl_2_‐exchangeable P was quantified (American Public Health Association [APHA] et al., 2017).

### 3.4 Statistical methods

We checked for correlations between SS concentrations and P fractionation of SS using Spearman's rho. We analyzed SS, TP, and DRP hysteresis loops for each event and classified events as clockwise or counterclockwise (Figures S2–S7; C. Evans & Davies, 1998). All statistical analyses were run in R version 4.4.1 (R Core Team, 2024) with *α* = 0.05 to assess statistical significance.

## 4 RESULTS AND DISCUSSION

### 4.1 Yields of phosphorus and SS

Most TP leaving the field was DRP during 2022; at the stream only half of the TP annual load was DRP. Total SS, TP, and DRP yields in 2022 in East River were 215 kg·ha^−1^·year^−1^, 0.81 kg P·ha^−1^·year^−1^, and 0.41 kg P·ha^−1^·year^−1^, respectively (Table S1), which was about half of the mean annual yields measured in previous years. The TP yield of 6.94 kg P·ha^−1^·year^−1^ at the field site was similar to the mean annual yield from 2012 to 2021, but the SS yield of 84 kg·ha^−1^·year^−1^ was the lowest recorded annual yield. The DRP yield of 5.94 kg P·ha^−1^·year^−1^ was the second highest recorded annual yield for the field (Table S1). The DRP:TP at the field site in 2022 was the highest for the period of record and much higher than the mean annual DRP:TP of 0.32. This indicates that our sampling occurred during a year that had a higher proportion of DRP than PP relative to previous years.

Many conservation practices were implemented across the East River Basin in 2016 and 2017 (Merriman et al., 2019) with cover crops planted at the field site starting in November 2016 (Hanrahan et al., in press). After implementation, event DRP yield, surface runoff volume (mm), and peak discharge (m^3^ s^−1^) increased at the field, whereas SS yield decreased (Hanrahan et al., in press). Mean annual SS, TP, and DRP yields at the field before implementation were 3439 kg·ha^−1^·year^−1^, 6.41 kg P·ha^−1^·year^−1^, and 0.43 kg P·ha^−1^·year^−1^, respectively, whereas after implementation mean annual SS, TP, and DRP yields were 1484 kg·ha^−1^·year^−1^, 7.18 kg P·ha^−1^·year^−1^, and 3.57 kg P·ha^−1^·year^−1^, respectively. At the East River site, mean annual SS, TP, and DRP yields increased after implementation of practices. Mean annual SS, TP, and DRP yields for the upper East River during 2012–2016 were 364 kg·ha^−1^·year^−1^, 1.14 kg P·ha^−1^·year^−1^, and 0.51 kg P·ha^−1^·year^−1^, respectively, while during 2017–2022, mean annual yields were 540 kg·ha^−1^·year^−1^, 1.95 kg P·ha^−1^·year^−1^, and 0.93 kg P·ha^−1^·year^−1^, respectively. During the later period, mean discharge in the East River also increased from 0.77 to 1.19 m^3^ s^−1^, which may have increased yields.

### 4.2 Seasonal and management effects on surface runoff and yields

**FIGURE 2:**
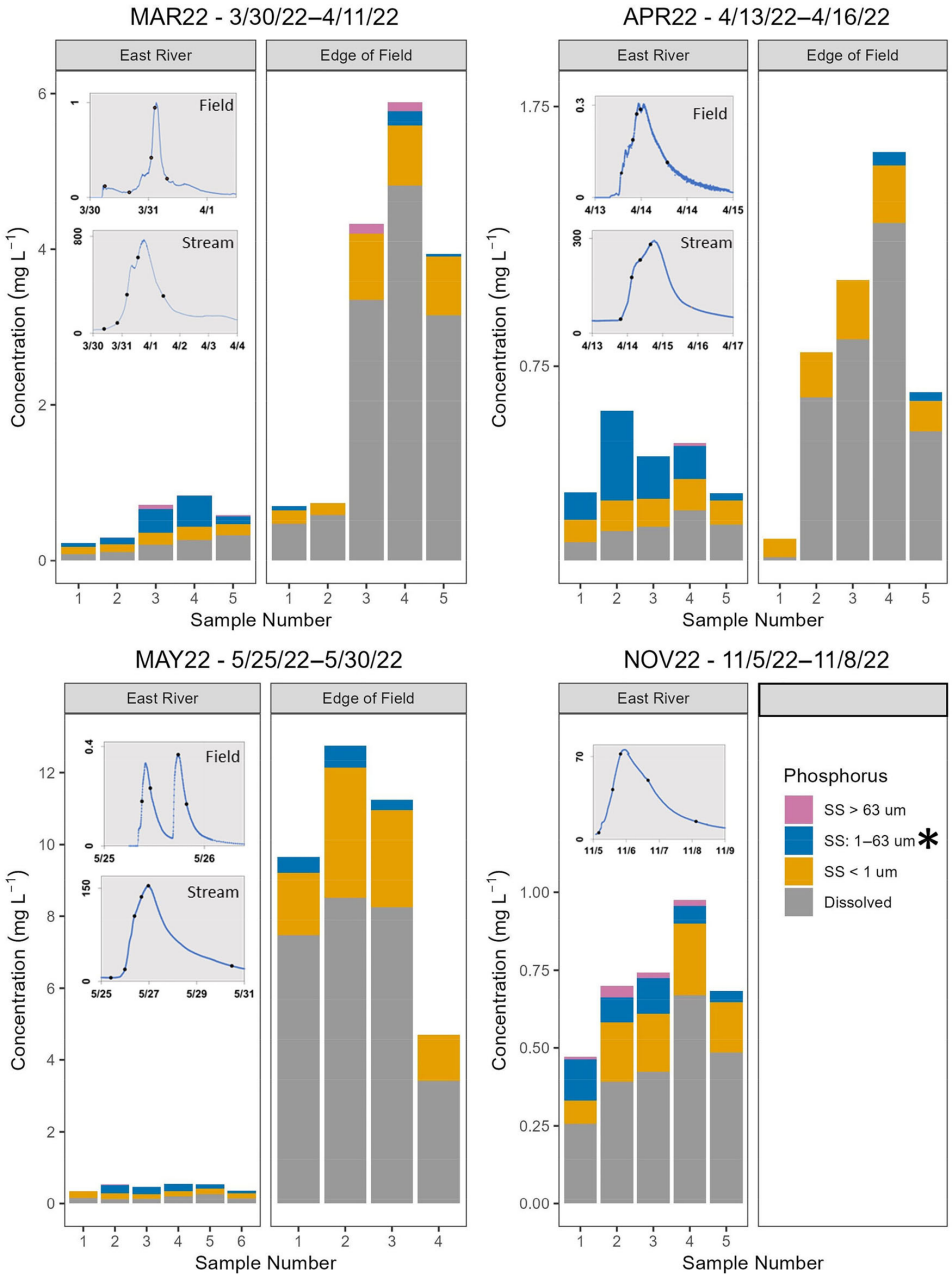
Dissolved phosphorus concentrations in stream water and phosphorus concentrations in different sediment particle size fractions of suspended sediment (SS) during surface runoff events that occurred at the farm field and East River sites in 2022. There was no surface runoff from the field during the NOV22 event. The asterisk indicates predominant size fraction for all events. Note difference in scales for concentrations and streamflow. Inset graphs display when samples were collected along the hydrograph, and *y*‐axis depicts streamflow in ft^3^·s^−1^.

**FIGURE 3:**
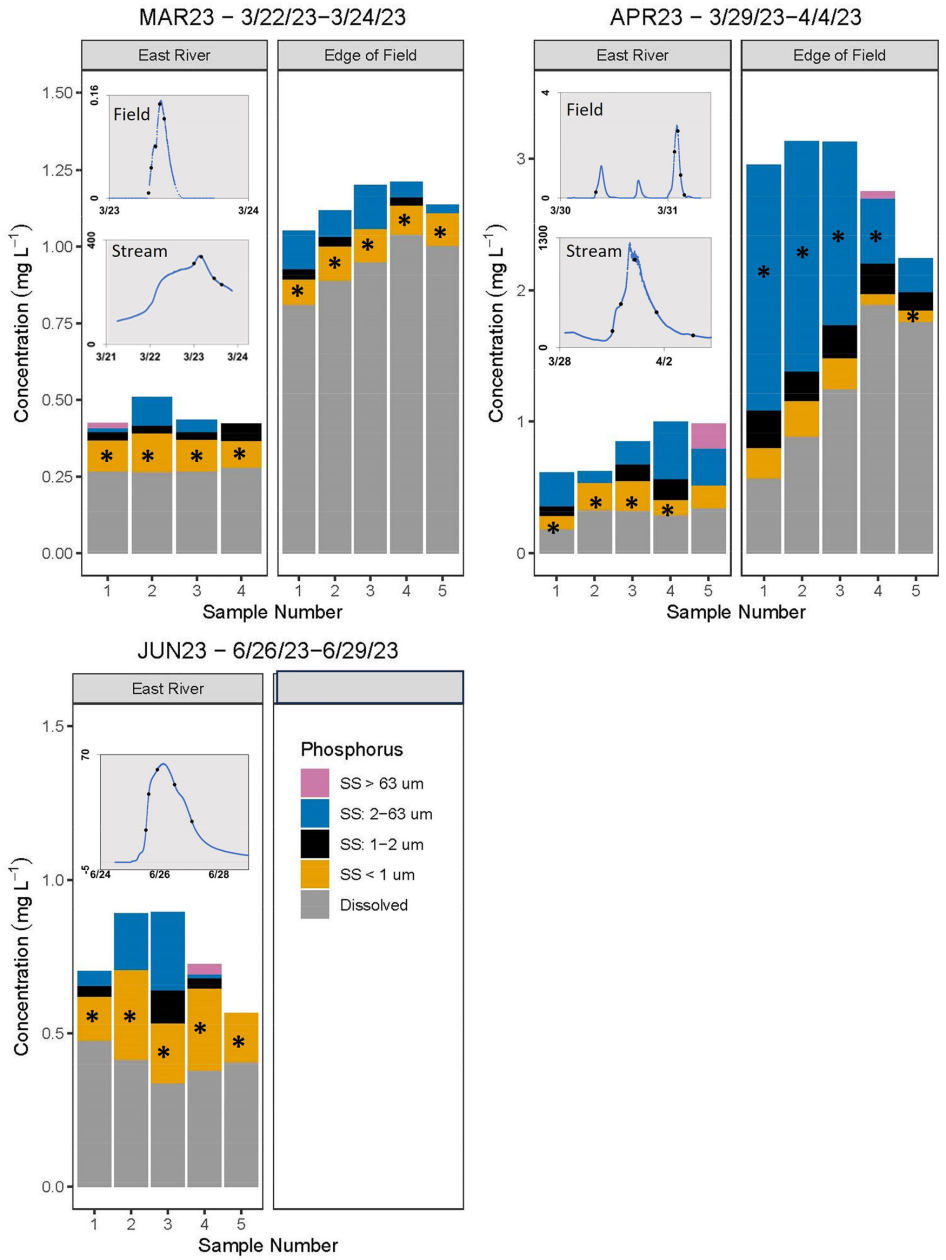
Dissolved phosphorus concentrations in stream water and phosphorus concentrations in different sediment particle size fractions of suspended sediment (SS) during surface runoff events that occurred at the farm field and East River sites in 2023. Surface runoff was not measured at the field during the JUN23 event. The asterisk indicates predominant size fraction for each event if there was one. Note difference in scales for concentrations and streamflow and addition of the 1‐ to 2‐µm size class in 2023. Inset graphs display when samples were collected along the hydrograph, and *y*‐axis depicts streamflow in ft^3^·s^−1^.

The two largest surface runoff events occurred in late March and early April of both years. The largest surface runoff event was MAR22, driven by the highest rainfall total and longest duration, with the highest TP and DRP yields from the field, but not the highest SS yield (Table 1; Figure 2). The ground was frozen during this event. The second largest surface runoff event was APR23, which occurred 4 days after 254 mm of snow fell and 5 days after the smallest surface runoff event at the field (MAR23). Rainfall total, surface runoff volume from the field, and surface runoff event duration were lower in APR23 compared to MAR22, but SS yields and concentrations (Figure 1c) were higher during APR23 (Table 1). The SS load in the East River during APR23 was twice the SS load during MAR22, and APR23 was the only event where the hysteresis pattern for SS was counterclockwise indicating more distal sources of SS (Figure S5). SS concentrations were over an order of magnitude higher at the field compared to the other events (Figure 1c), resulting in APR23 being the only event when the majority of TP leaving the field was PP (Figure 3). The cover crop was not successful in winter 2022/2023, which may have contributed to the higher SS concentrations. The hysteresis pattern for TP at the field was clockwise for APR23 (Figure S3) indicating concentrations were higher on the rising limb of the hydrograph, but during the descending limb, most P was dissolved indicating that after the initial flush of soil and PP off the field, more DRP was carried with storm water. The implication is that as the event receded, erosion from surface runoff on exposed soil decreased, and near‐surface movement of soil water resulted in exfiltration near the base of the steeply sloped field (Kirkby & Chorley, 1967; Montgomery et al., 1997).

**TABLE 1:** Information about surface runoff events, including yields of suspended sediment (SS), total phosphorus (TP), and dissolved reactive phosphorus (DRP), DRP to TP ratio, and median concentrations of SS‐normalized particulate P (P_ss_) sampled at the farm field and East River sites. Rainfall was measured at the East River site. Snowmelt estimates were estimated using the Snow Data Assimilation System (Barrett, 2003) and are reported in water equivalent values. Total rainfall amounts are rainfall and water equivalent values from snowmelt. Runoff is total amount of surface water leaving the field site during the event.

			Field site						East River				
Event	Dates	Rainfall [snowmelt] (mm)	Runoff (mm)	SS (kg·ha^−1^)	TP (kg·ha^−1^)	DRP (kg·ha^−1^)	DRP: TP	P_ss_ (ppm)	SS (kg·ha^−1^)	TP (kg·ha^−1^)	DRP (kg·ha^−1^)	DRP:TP	P_ss_ (ppm)
MAR22	3/30/2022–4/11/2022	75 [23]	79	10.73	2.546	2.293	0.90	39,450	87.29	0.245	0.115	0.47	1708
APR22	4/13/2022–4/16/2022	15	11	0.51	0.116	0.100	0.88	32,529	13.93	0.037	0.015	0.41	1331
MAY22	5/25/2022–5/30/2022	36	4	2.71	0.422	0.355	0.84	48,994	8.32	0.035	0.019	0.54	1986
NOV22	11/5/2022–11/8/2022	20	0	0	0	0		0	1.23	0.020	0.014	0.73	4203
MAR23	3/23/2023–3/24/2023	16 [16]	1	0.62	0.014	0.013	1.00	3354	11.29	0.052	0.032	0.61	2387
APR23	3/29/2023–4/4/2023	104 [58]	21	403.52	1.177	0.228	0.19	1543	243.92	0.493	0.138	0.28	917
JUN23	6/26/2023–6/29/2023	16	0	0	0	0		0	2.84	0.019	0.011	0.59	3299

In agricultural areas where frozen soil and snow cover are common in winter, surface runoff from farm fields typically peaks during snowmelt and when the soil is thawing in late winter‐early spring (Van Esbroeck et al., 2016; Williamson et al., 2023). Increased surface runoff coincides with higher streamflow and in‐stream SS and P loads compared to other times of the year (Dolph et al., 2019; Good et al., 2019). Streamflow was elevated in the East River for most of March 2023. The snowmelt event (MAR23) 5 days prior to APR23 had minimal surface runoff from the field indicating that most melted snow infiltrated into the soil, increasing soil‐water content and subsequent hydrologic response (Williamson et al., 2023). The day after MAR23 ended, 254 mm of snow fell, and during the 7 days of APR23, 46 mm of rain fell. The precipitation melted the snow, likely saturating the field, causing soil erosion on a field that was mostly bare soil; these conditions resulted in extremely high SS loads that were transported from the field (Liu et al., 2013).

Although SS in surface runoff from the field was relatively low during MAR22, TP and DRP loads were the highest recorded during the study. On‐field management practices, including establishment of cover crops, may have contributed to high DRP in surface runoff. An assessment of surface runoff before and after implementation of conservation practices in November 2016 found an increase in DRP concentrations and yields after implementation (Hanrahan et al., in press). Surface applying manure and chemical fertilizer without incorporating it into the soil profile can also lead to elevated DRP in surface runoff (Macrae et al., 2021). Surface application of manure occurred three times between November 2021 and November 2022 (Gierke et al., 2025). Conservation cover crops, which were planted on the field during both winters of the study but only really established in the first winter, reduce soil erosion, but DRP can be released from the vegetation during freeze‐thaw cycles (Kieta et al., 2018). The field had not been tilled since May 2020; conservation tillage reduces soil erosion but can increase the labile P fraction that is at the soil surface, increasing the amount of DRP leaving the field (Jarvie et al., 2017; Macrae et al., 2021).

The highest concentrations of TP and DRP in surface runoff were in late May during MAY22 (Figure 2), which occurred 7 days after the field was planted and fertilized with manure and chemical fertilizers (Figure 1b). MAY22 was the only event where a clockwise hysteresis pattern was observed for DRP at the field (Figure S4). A clockwise hysteresis pattern was also observed for TP (Figure S3), indicating that soil water that contained high DRP concentrations was flushed during the rising limb of the hydrograph. Repeated manure applications can cause a buildup of residual P in the soil that can be released during surface runoff events (Hanrahan et al., 2019). Events that occur as much as 3 weeks after a field has been fertilized are at higher risk of elevated P in runoff (Sharpley et al., 2001) with risk greatest within the first week (D. R. Smith et al., 2007). Mean soil test P concentrations on the field were 80 mg P·kg^−1^ soil (Meyers et al., 2021), which exceeds the recommended range of 30–50 mg P·kg^−1^ soil (USDA Natural Resources Conservation Service [USDA‐NRCS], 2015).

### 4.3 Different phosphorus forms in field and stream

The P dynamics were different in the stream compared to field surface runoff. As hypothesized, DRP:TP was different, with concentrations of both TP and DRP higher at the field, and field DRP:TP generally >0.8 (excluding the APR23 event; Table 1). In contrast, more P in the stream was PP (Figures 2 and 3), with DRP:TP of 0.28–0.73. Magnitude and variability in TP across events were greater at the field. The TP concentrations in event samples in East River ranged from 0.23 to 1.00 mg P L^−1^, whereas median TP concentrations at the field ranged from 0.80 to 10.45 mg P L^−1^.

We had hypothesized that most PP, regardless of location, would be sorbed to fine clay (<1 µm) in SS, which was observed during some events. During three events in 2022, most PP at the field was present in the fine clay fraction (Figure 2), corresponding to the predominant particle fraction leaving the field (median: 90% of SS; range: 74%–100%; Gierke et al., 2025). In 2023, most PP leaving the field was in the silt fraction, especially during APR23 (Figure 3) when most SS was silt (median: 53% of SS; range 30%–83%). This difference in particle‐size fractionation between the 2 years corresponded to an order of magnitude difference in median concentration of SS‐normalized PP (P_ss_) with median P_ss_ concentrations in 2022 much higher than those during 2023 events when more SS was transported (Table 1). In other studies, the particle size distribution of SS leaving tilled corn fields was 0.05–100 µm (Panuska et al., 2011; Poirier et al., 2012), with clay particles being more enriched with P than silt and sand (Panuska et al., 2011). Smaller particles have more surface area for the same mass and are preferentially removed during surface runoff events (Sharpley et al., 2013).

During the first three events in 2022, most PP in the stream was sorbed to silt and coarse clay even though fine clay was the largest proportion of SS (median: 72% of SS; range: 53%–100%). As SS concentrations increased in the East River, PP in the silt and coarse clay fraction also increased (*ρ* = 0.78, *p* < 0.001; Figure S8). In 2023, when streamflow was higher, more P was present in the silt and fine clay fractions (Figure 3). Rarely was P present in the sand fraction at either location (Figures 2 and 3); little sand was transported during events (median: 0% of SS; range: 0%–17%). The SS source in streams can play an important role in particle size and PP concentrations. Streambank erosion accounts for more than half of SS loads in many agricultural streams in the Great Lakes Basin (Fitzpatrick et al., 2019; Williamson et al., 2020). Streambank sediment can be similarly P‐enriched to cropland sources of SS (Williamson et al., 2020, 2024). Fingerprinting of East River SS from 2022 identified in‐channel sources, including streambank and remobilized streambed sediment, as the largest contributors, with SS enriched in P compared to both in‐channel and upland sources (Williamson et al., in press).

Both SS and TP in stream water were lower during summer baseflow conditions and smaller surface runoff events, but the abundance of PP with SS was higher, indicating higher P_ss_. The DRP:TP of samples collected during baseflow in August 2022 were 0.9 and 0.78, respectively. Most stream‐water TP was DRP during NOV22 (Figure 2) and JUN23 (Figure 3); yet SS was more P‐enriched compared to other events (>3000 ppm P; Table 1). These events had the lowest streamflow (Figure 1b). Baseflow and lower streamflow facilitate P‐enrichment in SS (Owens & Walling, 2002; Pacini & Gächter, 1999; Williamson et al., 2023). The SS collected during NOV22 had the highest P_ss_. SS resuspended from streambed in the fall has been accumulating P all summer, resulting in higher P_SS_ (House et al., 1998). Sediment takes multiple events to move completely through the river network and can adsorb P as it is stored and remobilized (Verhoff et al., 1979). Bioavailable, MgCl_2_‐exchangeable P concentration in the SS in October and November was 196.5 mg P·kg^−1^ dry sediment, which was much higher than the range of 20.2–36.3 mg P·kg^−1^ dry sediment observed from April through September. MgCl_2_‐exchangeable P is the pool of sorbed P that is readily available for release to stream water indicating that although less PP is transported during fall, the potential for bioavailable P release could be similar to or higher than when more DRP is transported.

**FIGURE 4:**
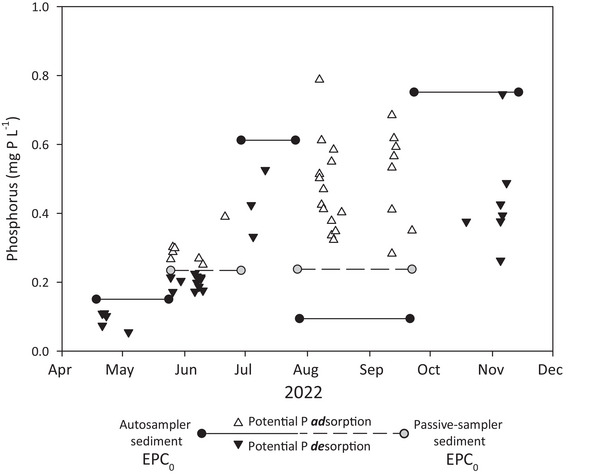
Equilibrium phosphorus concentrations (EPC_0_) of stream suspended sediment from monthly integrated samples collected in the East River during 2022. Triangles depict dissolved reactive phosphorus (DRP) concentrations of water samples collected during the study. Orientation of triangle indicates potential adsorption or desorption of DRP from the sediment.

The difference in P forms between field and stream may reflect multiple drivers. First, P may spiral between DRP and PP as it progresses from field to stream (Withers & Jarvie, 2008). During all events except APR23, SS concentrations were higher in the stream (Figure 1c), which translates to more potential binding sites for adsorption of DRP (W. M. King et al., 2022). The potential for SS to adsorb P is based on availability of reactive metal hydroxide sorption sites on SS (D. J. Evans et al., 2004; House et al., 1998). Although we did not measure sorption sites of SS, we did measure potential of SS to de/sorb P in the stream. The capacity for P sorption varied during 2022. The EPC_0_ of SS collected throughout 2022 ranged from 0.094 to 0.75 mg P L^−1^ with highest EPC_0_ in July, October, and November (Figure 4). Comparing the EPC_0_ values to DRP concentrations of all baseflow and event samples collected in the East River during 2022 (U.S. Geological Survey, 2024) indicated that SS was potentially adsorbing P during the MAY22 event, in late June, and during August and September (Figure 4) and potentially desorbing P the rest of the time. The sorption potential can change during events based on changing DRP concentrations in stream water. The two lowest EPC_0_ of the SS were measured during spring and late summer indicating that SS had a higher capacity to sorb P during this time. More reactive iron‐sorption sites could be available on SS during early spring surface runoff events and late summer events that occur after prolonged periods of baseflow (Rosenberg & Schroth, 2017).

The difference in P forms also may be influenced by changing P dynamics on the field. Hanrahan et al. (in press) assessed conservation practice effects on nutrient runoff on our field and four other fields in the East River Basin and found similar effects of conservation implementation on P loads and yields across sites. In years prior to this study, more P was exported from the field as PP compared to DRP (Table S1) as a result of relatively high soil erosion. With conservation practice implementation, the amount and relative proportion of DRP leaving the field increased (Hanrahan et al., in press). Difference in spatial scales between the small field and stream can lead to a lagged effect of observing on‐field changes in nutrient dynamics in downstream receiving waters (Sharpley et al., 2013; D. R. Smith et al., 2019). The stream site integrates dynamic processes occurring across the entire basin, including instream P cycling (Ensign & Doyle, 2006), which may mask and delay changes observable at the field scale (Sharpley et al., 2013; D. R. Smith et al., 2019).

### 4.4 Bioavailability of PP

Finer particle fractions commonly have more bioavailable P. More bioavailable P was found in the <1 µm (fine clay; Poirer et al., 2012), <2 µm (clay; Stone & English, 1993), and <8 µm (fine silt + clay; Yao et al., 2016) fractions compared to the larger sized particle fractions in previous studies. Much of the bioavailable P was labile organic PP and P bound to metal hydroxides present on SS (Pacini & Gächter, 1999; Stone & English, 1993; Yao et al., 2016). The implication is that most PP in the East River, which was concentrated in the fine‐clay fraction, is likely to be bioavailable, with potentially less bioavailable P during larger events like APR23 during which silt carried more PP.

While most P leaving the field was DRP, it is possible that not all DRP was bioavailable. Some of the DRP could have been colloidal P, which is P that is bound to nanoparticles (1 nm–1 µm; Gu et al., 2020) and is PP. These colloidal particles could have passed through our 0.45‐µm filter. As much as 50% of DRP can be P bound to nanoparticles (Gu et al., 2020; River & Richardson, 2019). Colloidal P is associated with organic carbon (C) and iron‐oxyhydroxides (Gu et al., 2020). Bioavailability of colloidal P is influenced by the P‐to‐iron ratio of particles (Baken, Moens, et al., 2016) and stream water (Baken, Regelink, et al., 2016) and pH of stream water (Liang et al., 2010).

## 5 CONCLUSIONS

Events highlighted effects of recent weather and management on P in surface runoff. Field management focused on improving soil health and decreasing SS and PP transport from the field, but an unintended by‐product of management was increased DRP. Consequently, there is a management trade‐off between reducing soil erosion and unintentionally increasing the bioavailable portion of TP in surface runoff. Our analysis showed that P‐form dynamics continue after SS and P leave the field, where potential for DRP to sorb to SS results in enriched P_SS_. This highlights how integrated basin management that considers what happens as runoff leaves the field and continues through the stream may identify new methods of mitigating conditions that foster HABs.

HABs may persist in Green Bay even if TP is reduced below the Fox River total maximum daily load limit due to a larger bioavailable P component. Localized benefits of on‐field management may not reflect ongoing transport, de/sorption, and P storage downstream in stream‐channel sediment and water bodies. This information would be useful for watershed managers who are implementing plans to reduce SS and TP loads, with the understanding that although TP loads are reduced, bioavailability of P may increase with some management actions.

## AUTHOR CONTRIBUTIONS

**Rebecca M. Kreiling**: Conceptualization; data curation; formal analysis; funding acquisition; investigation; methodology; project administration; resources; software; supervision; validation; visualization; writing—original draft; writing—review and editing. **Tanja N. Williamson**: Conceptualization; funding acquisition; investigation; methodology; project administration; resources; supervision; visualization; writing—review and editing. **Faith A. Fitzpatrick**: Conceptualization; funding acquisition; investigation; project administration; supervision; writing—review and editing. **Kenna J. Gierke**: Data curation; formal analysis; investigation; methodology; writing—review and editing. **James D. Blount**: Conceptualization; data curation; funding acquisition; methodology; project administration; writing—review and editing. **Patrik M. Perner**: Data curation; methodology; writing—review and editing. **Isaac J. Mevis**: Data curation; methodology; project administration; writing—review and editing. **Heidi M. Broerman**: Data curation; investigation; methodology; project administration; visualization; writing—review and editing. **Katherine R. Merriman**: Conceptualization; funding acquisition; investigation; methodology; writing—review and editing. **Matthew J. Komiskey**: Conceptualization; funding acquisition; project administration; resources; supervision; writing—review and editing.

## CONFLICT OF INTEREST STATEMENT

The authors declare no conflicts of interest.

## Supporting information



The supplementary material includes a table of the annual yields of suspended sediment, total phosphorus, and soluble reactive phosphorus from the field site (2014‐2022) and the East River site (2012‐2022). Additional figures are included that illustrate the study area, the relation between suspended sediment and phosphorus, and the hysteresis patterns for suspended sediment, total phosphorus, and soluble reactive phosphorus at the field and East River sites for all events.
