# Supplementary material for: Changing dynamic phosphorus forms from field to stream during surface runoff events
Source: J Environ Qual. 2025 Dec 17;55(1):e70096. doi: 10.1002/jeq2.70096 (PMC12710158; doi:10.1002/jeq2.70096)
Supplement: Supplementary file 1 — The supplementary material includes a table of the annual yields of suspended sediment, total phosphorus, and soluble reactive phosphorus from the field site (2014‐2022) and the East River site (2012‐2022). Additional figures are included that illustrate the study area, the relation between suspended sediment and phosphorus, and the hysteresis patterns for suspended sediment, total phosphorus, and soluble reactive phosphorus at the field and East River sites for all events. [file JEQ2-55-0-s001.docx]

**SUPPLEMENTAL MATERIAL**

**Changing DYNAMIC phosphorus forms from field to stream during SURFACE runoff events**

Rebecca M. Kreiling^1*^, Tanja N. Williamson^2^, Faith A. Fitzpatrick^3^, Kenna J. Gierke^1^, James D. Blount^4^, Patrik M. Perner^5^, Isaac J. Mevis^6^, Heidi M. Broerman^3^, Katherine R. Merriman^7^ and Matthew J. Komiskey^3^

^1^U.S. Geological Survey, Upper Midwest Environmental Sciences Center, La Crosse, WI, USA

^2^U.S. Geological Survey, OH-KY-IN Water Science Center, Louisville, KY, USA

^3^U.S. Geological Survey, Upper Midwest Water Science Center, Madison, WI, USA

^4^U.S. Geological Survey, Upper Midwest Water Science Center, St. Paul, MN, USA

^5^U.S. Geological Survey, Upper Midwest Water Science Center, Rhinelander, WI, USA

^6^U.S. Geological Survey, Upper Midwest Water Science Center, Green Bay, WI, USA

^7^U.S. Geological Survey, New York Water Science Center, Troy, NY, USA

Tables: 1

Figures: 8

Supplemental Table S1. Annual (January through December) yields of suspended sediment (SS), total phosphorus (TP), and dissolved reactive phosphorus (DRP) and DRP to TP ratio of yields measured at the farm field and East River sites. Area-normalized yields are reported instead of loads to enable comparison between sites. Data collection at the field site began in 2014. Only surface runoff yields are reported.

|  | Field Site | | | | |  | | | East River | | | | |  | | |
| --- | --- | --- | --- | --- | --- | --- | --- | --- | --- | --- | --- | --- | --- | --- | --- | --- |
| Year | SS | TP | DRP | | DRP:TP | |  | SS | | TP | DRP | DRP:TP | | |  |  |
|  | kg·ha^-1^·yr^-1^ | | |  | | |  | kg·ha^-1^·yr^-1^ | | | | |  | | |  |
| 2012 |  |  |  | |  | |  | 340 | | 0.86 | 0.34 | 0.39 | | |  |  |
| 2013 |  |  |  | |  | |  | 218 | | 1.11 | 0.59 | 0.53 | | |  |  |
| 2014 | 5878 | 10.63 | 0.61 | | 0.06 | |  | 779 | | 1.62 | 0.55 | 0.34 | | |  |  |
| 2015 | 493 | 1.21 | 0.20 | | 0.16 | |  | 212 | | 0.95 | 0.51 | 0.54 | | |  |  |
| 2016 | 3947 | 7.39 | 0.50 | | 0.07 | |  | 273 | | 1.15 | 0.56 | 0.49 | | |  |  |
| 2017 | 1605 | 5.40 | 1.54 | | 0.28 | |  | 758 | | 1.96 | 0.93 | 0.48 | | |  |  |
| 2018 | 1822 | 10.84 | 6.47 | | 0.60 | |  | 547 | | 2.06 | 1.11 | 0.54 | | |  |  |
| 2019 | 2155 | 11.67 | 5.54 | | 0.48 | |  | 800 | | 3.31 | 1.84 | 0.56 | | |  |  |
| 2020 | 3082 | 6.71 | 0.95 | | 0.14 | |  | 729 | | 2.48 | 0.65 | 0.26 | | |  |  |
| 2021 | 159 | 1.52 | 0.95 | | 0.63 | |  | 194 | | 1.07 | 0.66 | 0.61 | | |  |  |
| 2022 | 84 | 6.94 | 5.94 | | 0.86 | |  | 215 | | 0.81 | 0.41 | 0.50 | | |  |  |


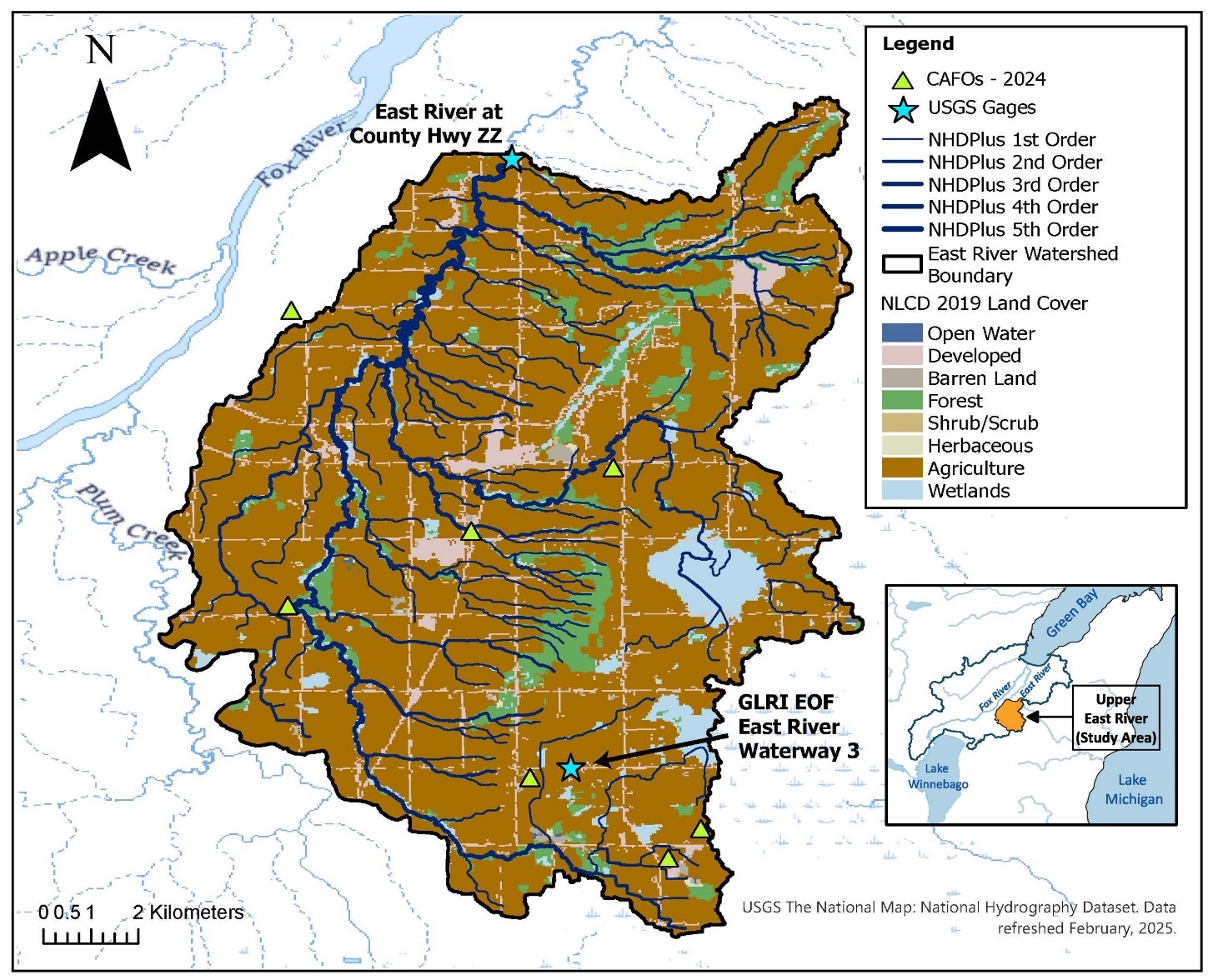


Supplemental Figure S1. Location of East River streamgage (USGS station 04085108), farm field site (USGS station 441520088045001), and confined animal feeding operations (CAFOs) in the upper East River basin. Land cover is 2019 National Land Cover Database (NLCD; Dewitz and USGS, 2024).


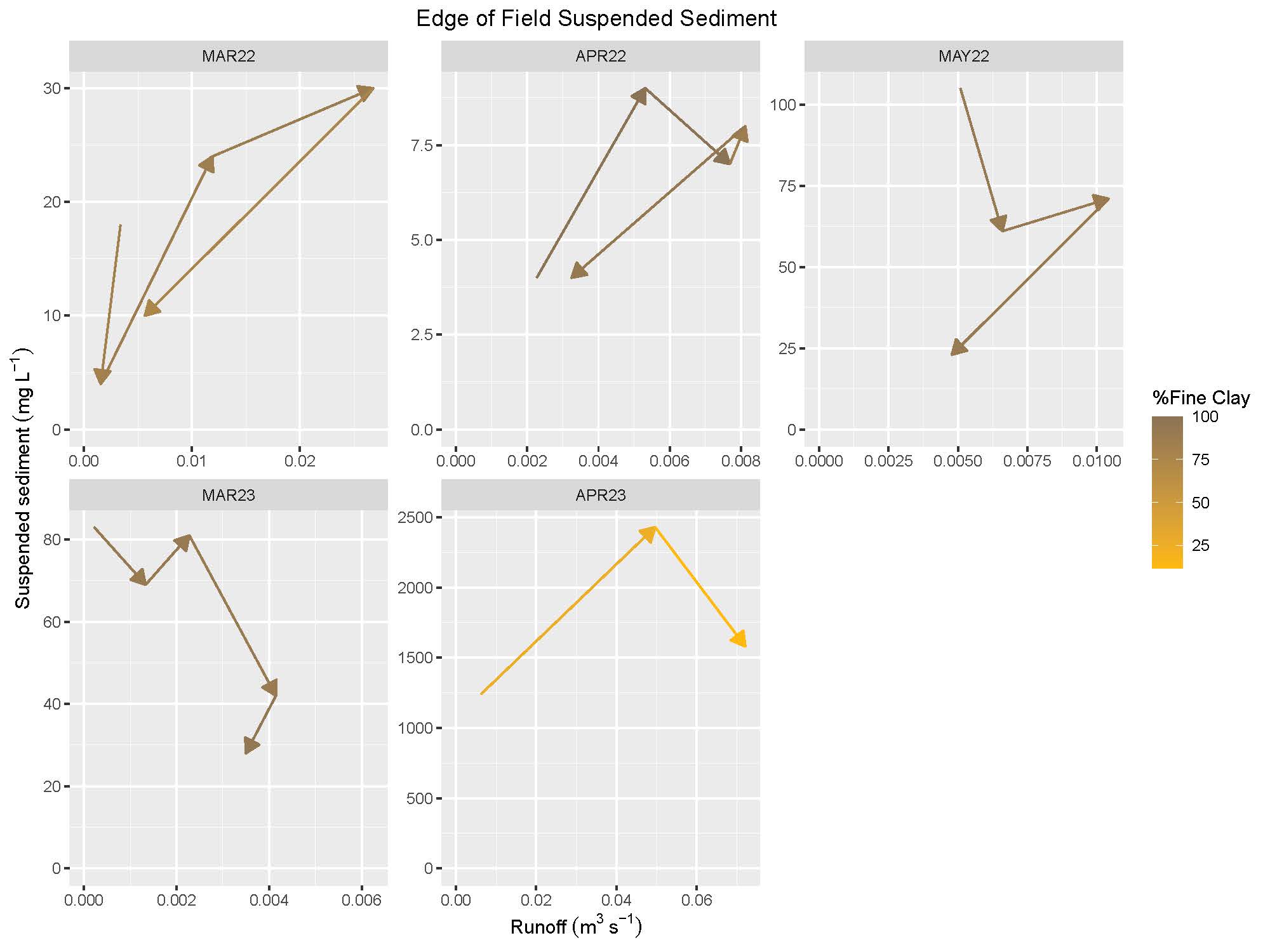


Supplemental Figure S2. Hysteresis loops for suspended sediment during events at the field site. Note that axis-scales vary among graphs.


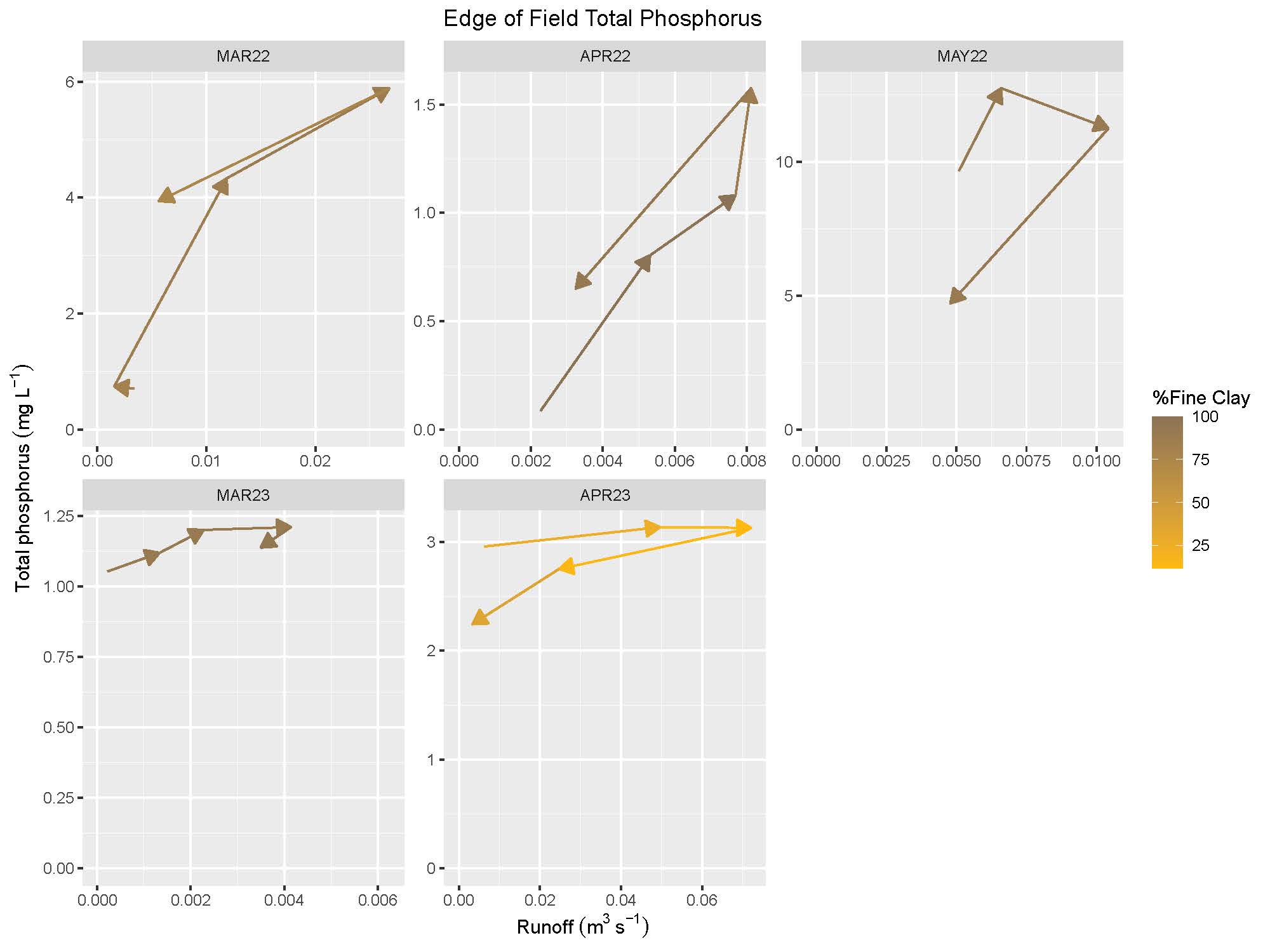


Supplemental Figure S3. Hysteresis loops for total phosphorus during events at the field site. Note that axis-scales vary among graphs.


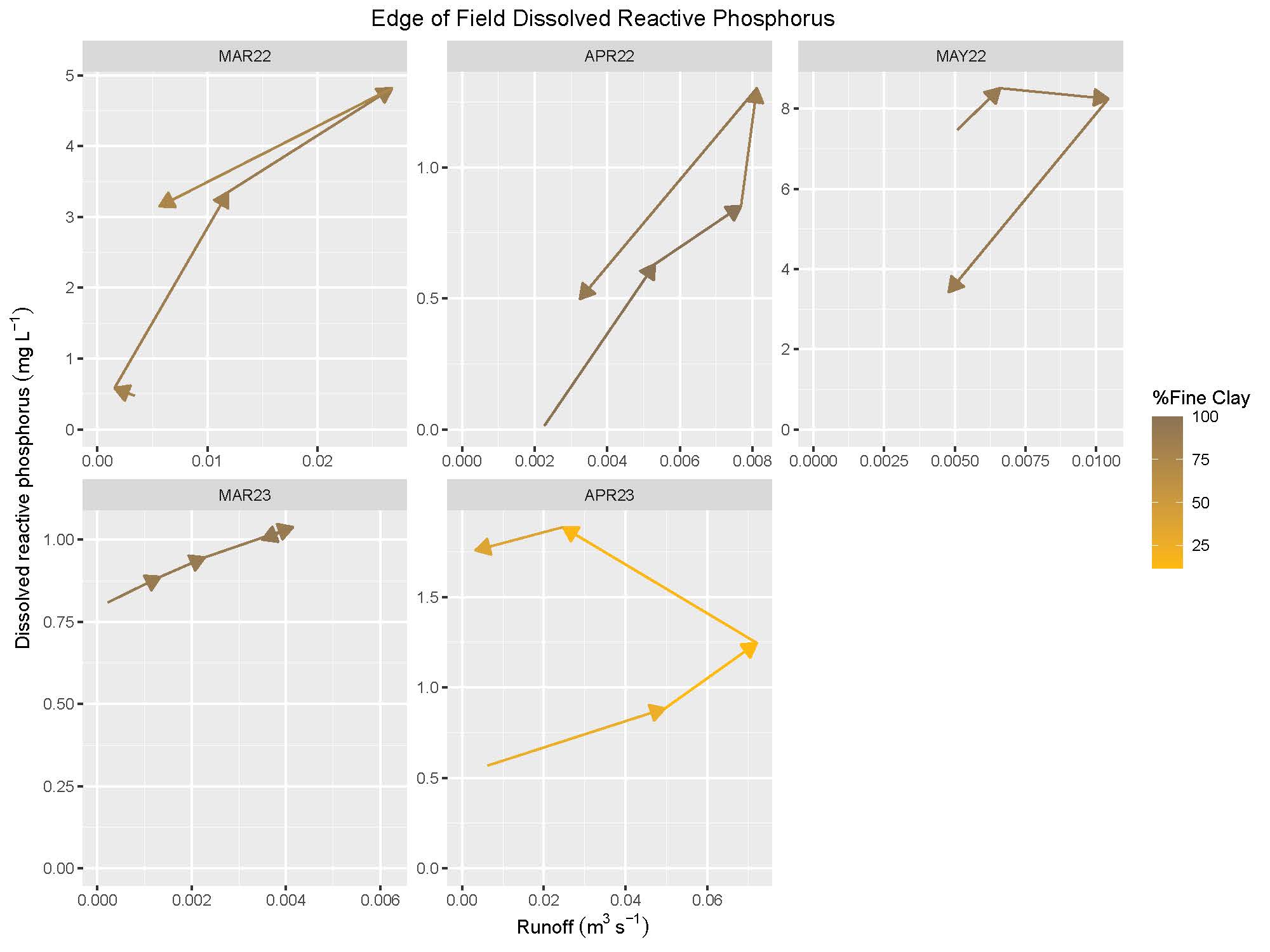


Supplemental Figure S4. Hysteresis loops for dissolved reactive phosphorus during events at the field site. Note that axis-scales vary among graphs.


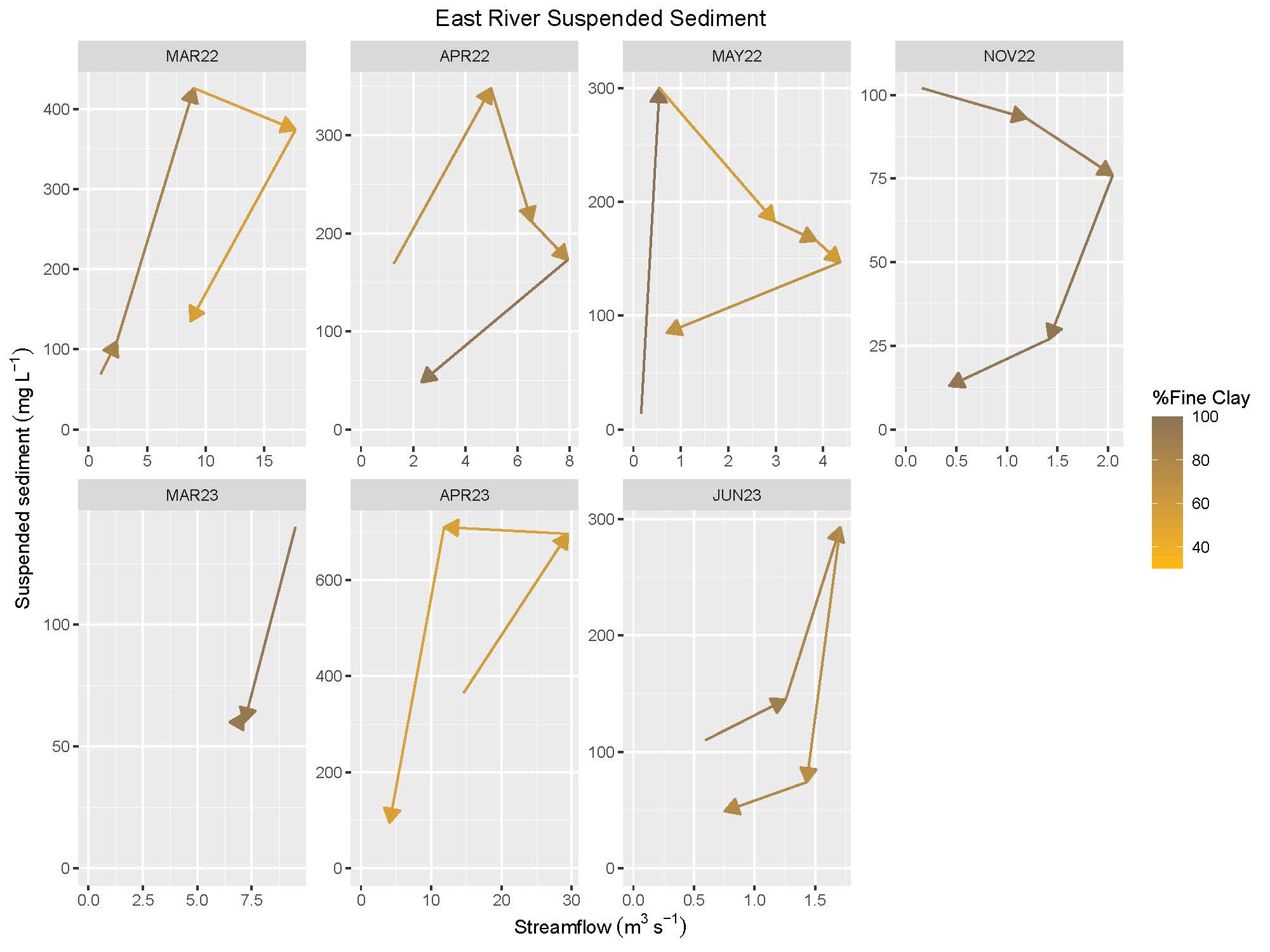


Supplemental Figure S5. Hysteresis loops for suspended sediment during events at the East River site. Note that axis-scales vary among graphs.


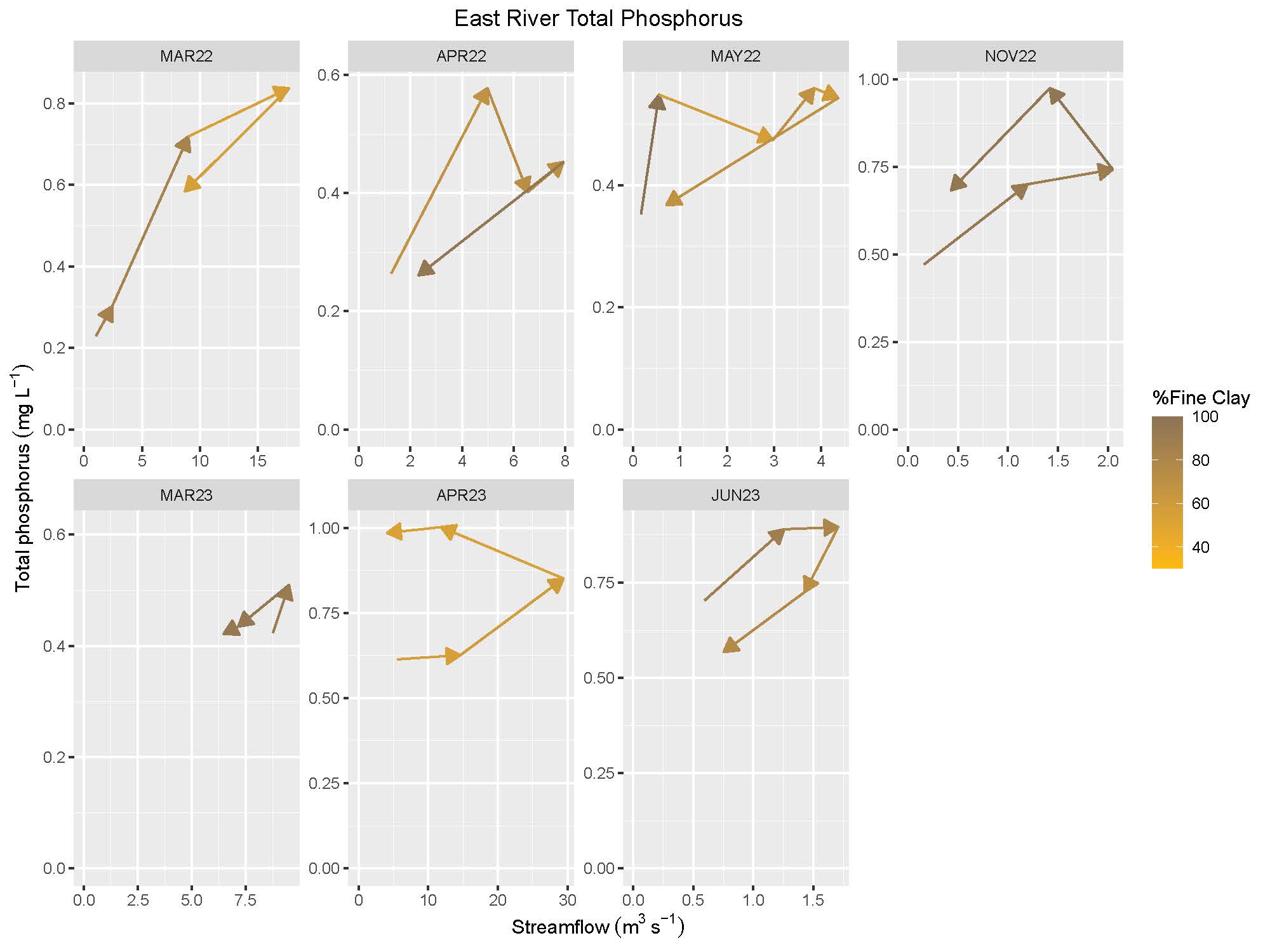


Supplemental Figure S6. Hysteresis loops for total phosphorus during events at the East River site. Note that axis-scales vary among graphs.


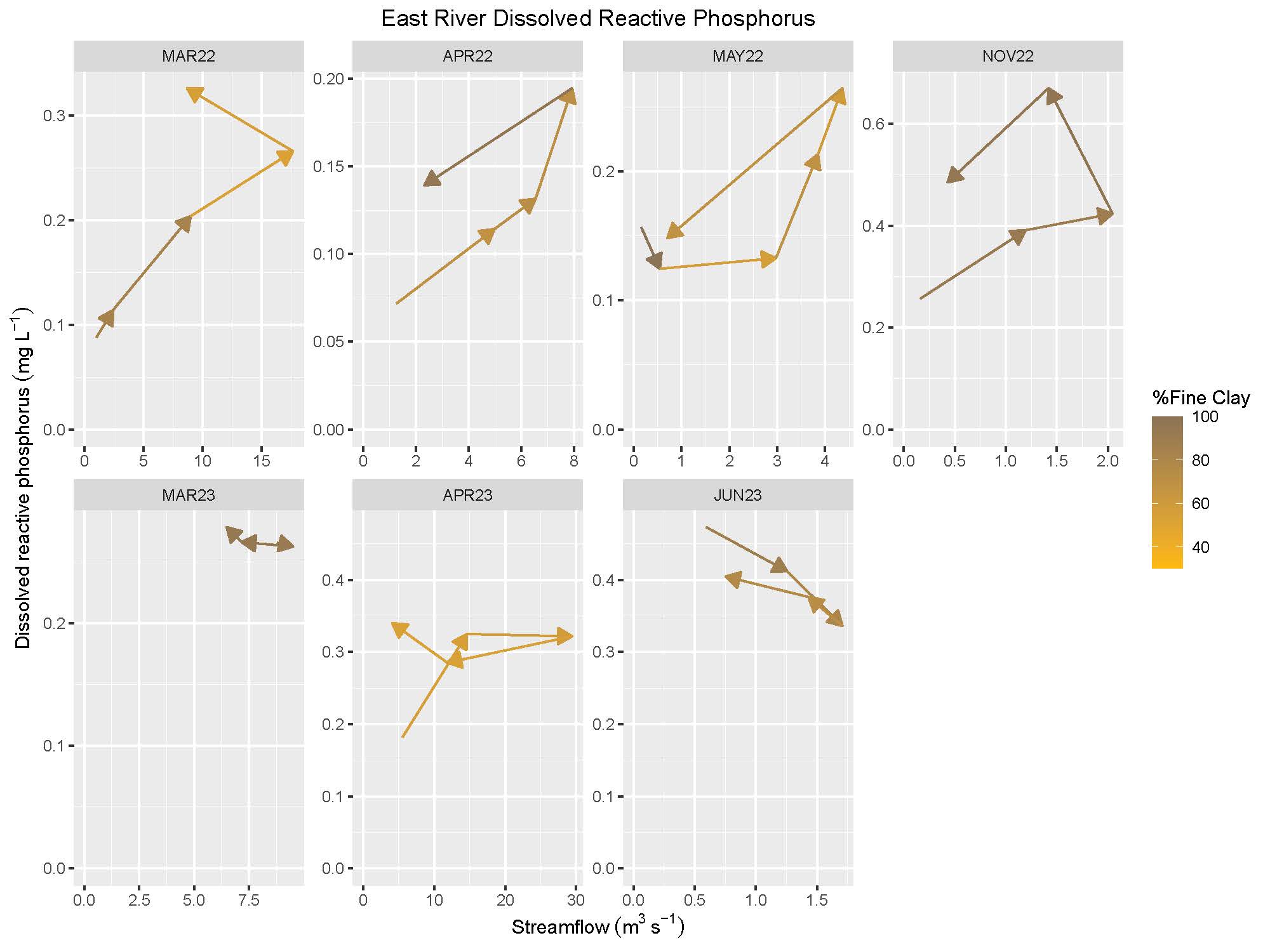


Supplemental Figure S7. Hysteresis loops for dissolved reactive phosphorus during events at the East River site. Note that axis-scales vary among graphs.


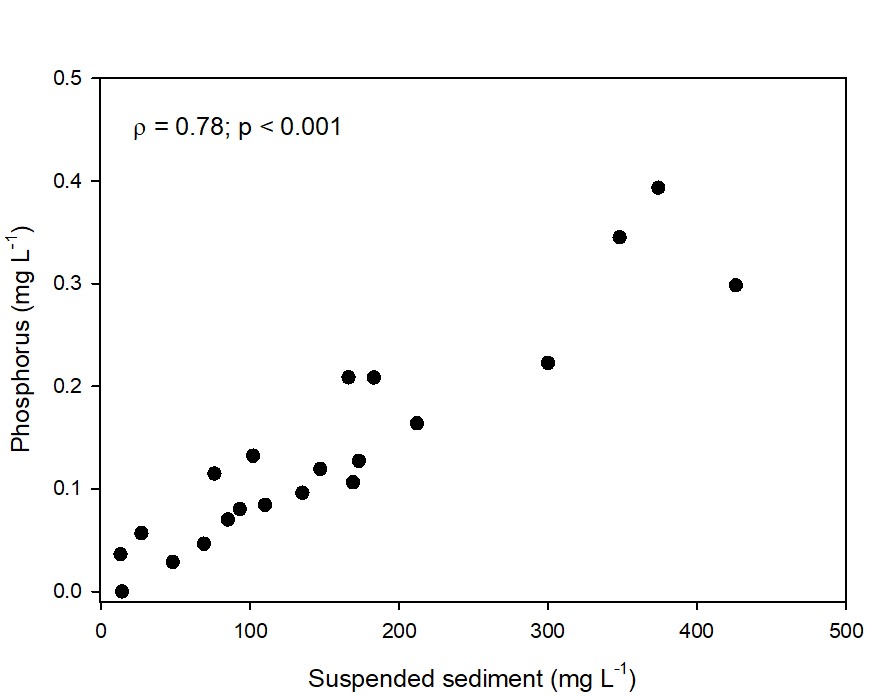


Supplemental Figure S8. Relation between suspended sediment concentration and particulate phosphorus concentration of the silt and coarse clay fraction (1-63 μm) from samples collected in the East River during runoff events in 2022.
